# Margination of Fluorescent Polylactic Acid–Polyaspartamide based Nanoparticles in Microcapillaries In Vitro: the Effect of Hematocrit and Pressure

**DOI:** 10.3390/molecules22111845

**Published:** 2017-10-28

**Authors:** Emanuela Fabiola Craparo, Rosa D’Apolito, Gaetano Giammona, Gennara Cavallaro, Giovanna Tomaiuolo

**Affiliations:** 1 Laboratory of Biocompatible Polymers, Dipartimento di Scienze e Tecnologie, Biologiche, Chimiche e Farmaceutiche (STEBICEF), Università di Palermo-via Archirafi, 32-90123 Palermo, Italy; emanuela.craparo@unipa.it (E.F.C.); gaetano.giammona@unipa.it (G.G.); 2 Dipartimento di Ingegneria Chimica, dei Materiali e della Produzione Industriale, Università di Napoli Federico II, P.le V. Tecchio 80, 80125 Napoli, Italy; rosa.dapolito@unina.it (R.D); g.tomaiuolo@unina.it (G.T.); 3 IBF-CNR, 90143 Palermo, Italy; 4 Mediterranean Center for Human Health Advanced Biotechnologies (CHAB), ATeNCenter, University of Palermo, 90100 Palermo, Italy; 5 CEINGE Biotecnologie avanzate, Via Gaetano Salvatore 486, 80145 Napoli, Italy

**Keywords:** α,β-poly-(N-2-hydroxyethyl)-d,l-aspartamide (PHEA), poly(lactic acid) (PLA), poly(ethylene glycol) (PEG), polymeric nanoparticles, margination

## Abstract

The last decade has seen the emergence of vascular-targeted drug delivery systems as a promising approach for the treatment of many diseases, such as cardiovascular diseases and cancer. In this field, one of the major challenges is carrier margination propensity (i.e., particle migration from blood flow to vessel walls); indeed, binding of these particles to targeted cells and tissues is only possible if there is direct carrier–wall interaction. Here, a microfluidic system mimicking the hydrodynamic conditions of human microcirculation in vitro is used to investigate the effect of red blood cells (RBCs) on a carrier margination in relation to RBC concentration (hematocrit) and pressure drop. As model drug carriers, fluorescent polymeric nanoparticles (FNPs) were chosen, which were obtained by using as starting material a pegylated polylactic acid–polyaspartamide copolymer. The latter was synthesized by derivatization of α,β-poly(N-2-hydroxyethyl)-d,l-aspartamide (PHEA) with Rhodamine (RhB), polylactic acid (PLA) and then poly(ethyleneglycol) (PEG) chains. It was found that the carrier concentration near the wall increases with increasing pressure drop, independently of RBC concentration, and that the tendency for FNP margination decreases with increasing hematocrit. This work highlights the importance of taking into account RBC–drug carrier interactions and physiological conditions in microcirculation when planning a drug delivery strategy based on systemically administered carriers.

## 1. Introduction

Nanomedicine holds great promises in the treatment of a wide range of diseases, such as cancer, pain, infections and inflammatory disorders [1,2]. In fact, the primary aim of nanomedicine is the improvement of human health delivering drug molecules within the body to their final biological target with minimal toxicity and in a controlled manner. For this purpose, the use of nano- and micro-particulate drug delivery systems has emerged as a valuable potential tool for performing the main goals of nanomedicine (e.g., targeted delivery, stability of the drug, high permeability, controlled release-kinetic and reduced side-effects) [2]. To evaluate the potential in delivery efficiency of drug carriers, it is crucial to study their transport, adhesion and distribution in blood flow. In particular, for particle transport and distribution in microcirculation (i.e., capillaries, arterioles and venules, where most of the exchange with tissues takes place), the particulate nature of blood and the deformability of RBCs needs to be considered [3,4]. A key step in particle-based drug delivery through microcirculation is particle migration from blood flow to vessel walls, also known as “margination”, which promotes particle contact and adhesion to the vessel wall. In physiology, the term refers to the flow behavior of white blood cells (WBCs) and platelets, which concentrate in the RBC-free-layer (RBC-FL), a near-wall region depleted of RBCs, which originates from the migration of RBCs toward the vessel centerline due to a hydrodynamic lift [5,6,7]. In analogy with platelets and WBCs, drug carriers within the bloodstream are also expected to migrate in the RBC-FL near the vessel wall [8,9,10]. The phenomenon of margination has been studied either by numerical simulations [8,11] or by experimental studies in vitro, the latter being focused on the dependence of micro-particles (μ-Ps) distribution and delivery efficacy on the presence of RBCs, shear rate, particle size, shape and surface charge [3,9,12,13]. It has been shown that margination, which is almost absent when particles are suspended in a cell-free medium, is drastically enhanced by RBCs, in a pressure drop-dependent manner. In particular, as the shear rate increases, the μ-P concentration near the wall increases. Margination is also affected by μ-P size and shape, larger spherical/discoid particles being more effectively marginated both in vitro and in vivo [14]. μ-Ps with different surface charge, instead, show a comparable margination propensity, suggesting that the presence of RBCs governs suspension flow behavior independently on μ-P surface charge [9]. Moreover, the chemical nature of μ-P could be another important aspect to take into account when designing drug delivery strategies. 

In this work, we use a microfluidic-based system to mimic in vitro the transport in microcapillaries of fluorescent polymeric nanoparticles (FNPs), chosen as model drug carriers, in order to investigate the effect of RBC volume fraction (i.e., hematocrit) and the imposed pressure drop on FNP margination. FNPs with nanometer and spherical size were obtained by following the high pressure homogenization (HPH) method, and by using as starting material a pegylated polylactic acid–polyaspartamide copolymer. The latter was obtained by derivatization of α,β-poly(N-2-hydroxyethyl)-d,l-aspartamide (PHEA) with Rhodamine (RhB), polylactic acid (PLA) and then poly(ethyleneglycol) (PEG) chains. The obtained FNPs also showed adequate physical-chemical properties to be used to investigate their behavior for in vitro margination experiments. 

Obtained results highlights the importance of taking into account the role of RBC–drug carrier interactions as well as all the parameters that can influence particles transport in microcirculation, in order to optimize drug delivery strategy for intravascular administration.

## 2. Results

### 2.1. Design of Fluorescent Polymeric Nanoparticles (FNPs) 

In this paper, a fluorescent drug carrier with proper size and surface characteristics was realized to investigate on the in vivo fate of nanostructured systems once administered via intravascular route. This aim was reached by using polymeric fluorescent nanoparticles (FNPs) with nanometric size, highly hydrophilic surface and low zeta potential, that were prepared by following a well-known method such as the high pressure homogenization (HPH) [15]. The FNPs were prepared using a derivative of α,β-poly(N-2-hydroxyethyl)-d,l-aspartamide (PHEA)as starting copolymer [16,17], which was functionalized in different steps with rhodamine B (RhB) moieties, polylactide (PLA) and polyethylene glycol (PEG) chains, to obtain PHEA-RhB-PLA-PEG copolymer, as described elsewhere [15]. The possibility to introduce a fluorescent probe permanently conjugated on the polymeric backbone gives a very high potential in imaging, with the possibility to follow the fate of either the copolymer or obtained copolymer-based nanostructured systems in vitro and in vivo [18]. Moreover, the functionalization reactions on PHEA with PLA and PEG have specific goals: the introduction on the main backbone of biodegradable hydrophobic chains to obtain an amphiphilic copolymer, and of biocompatible chains to increase the biocompatibility to the resulting copolymer and to confer stealth properties, respectively. 

These FNPs were chosen because their biocompatibility and their potential as drug delivery systems, by either systemic or pulmonary administrations, were already proven [18,19,20]. In particular, the absence of cytotoxicity of particles with similar composition and chemical-physical properties was evaluated in vitro on cancer cell lines such as human hepatocellular carcinoma cells (HepG2) and human cervical carcinoma cells (HeLa) after incubation for 72 h with FNPs dispersion at concentrations ranging between 0.1 and 0.5 mg/mL, and on human bronchial epithelial cells (16-HBE) after 24 h incubation at FNPs concentrations included between 0.01 and 1 mg/mL. Moreover, the absence of cell apoptosis and necrosis induction and of modification of the constitute expression of survivin on 16-HBE due to the presence of FNPs was also evaluated [20]. 

**Figure 1:**
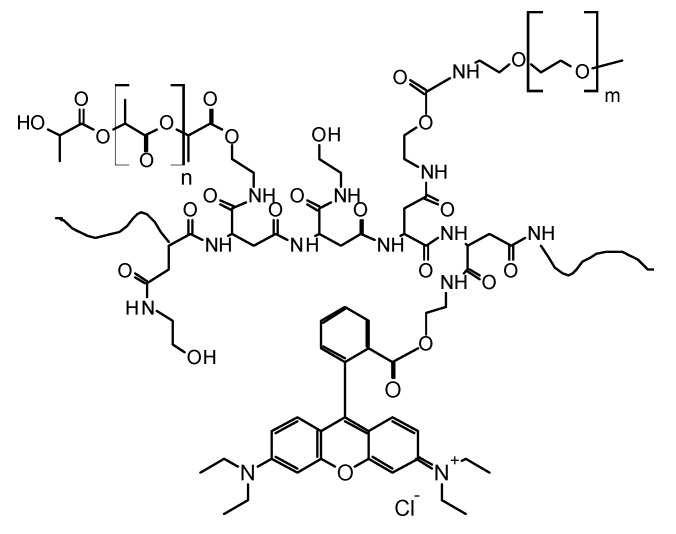
Chemical structure of PHEA-RhB-PLA-PEG copolymer (*m* = 44, *n* = 194).

The experimental conditions were properly chosen in order to modulate the Derivatization Degrees (DD) in RhB, PLA and PEG, to achieve a copolymer with proper structural and functional properties for obtaining a designed drug carrier [15]. In particular, the synthetic steps were carried out by following some functionalization reactions already standardized and reported in the literature, and the experimental conditions were chosen to achieve DD_RhB_, DD_PLA_ and DD_PEG_, respectively, equal to 0.61 mol %, 4.0 mol % and 2.0 mol % with respect to the moles of repeating units of PHEA, as determined by ^1^H-NMR analyses. The linkage of RhB, PLA and PEG to PHEA was also confirmed by SEC analysis, which revealed a weight average molecular weight (${\bar{M}}_{w}$) equal to 168.4 kDa (with ${\bar{M}}_{w}/{\bar{M}}_{n}$ = 1.45). The chemical structure of the PHEA-RhB-PLA-PEG copolymer is schematically shown in Figure 1.

FNPs were obtained by following a well-known method to produce polymeric and lipid nanoparticles such as the high pressure homogenization (HPH) and by using as starting material PHEA-RhB-PLA-PEG copolymer, avoiding the use of surfactant or other stabilizing agents thanks to the amphiphilic properties of the copolymer. Briefly, the process involved the preparation of an oil in water (o/w) emulsion by fast mixing the organic solution of the copolymer with bidistilled water, which, after a proper dilution with bidistilled water, was subjected to HPH (one cycle), then to the evaporation of organic solvent under reduced pressure, and finally the obtained FNPs were recovered as fluffy powder by freeze-drying in the presence of polyvinylpyrrolidone (PVP) as cryoprotectant. 

Once freeze-dried, the FNPs were re-dispersed in Dulbecco’s phosphate buffer saline (DPBS) and characterized in terms of mean number distribution size, polydispersity of distribution and ζ potential by using Photon Correlation Spectroscopy (PCS) to evaluate if possess effectively colloidal dimensions and adequate surface properties to be administered by intravenous route. It was found that these FNPs show nanoscaled size (594.2 ± 149.5 nm) and slightly negative zeta potential (−6.94 mV). The latter characteristic is due probably to the PEG chains into the starting material use to produce the nanoparticle that is preferentially exposed onto the nanoparticle surface, as already demonstrated in a previous paper [19]. However, FNP dispersion is physically stable within few hours; on the contrary, after about 24 h, particles aggregate and precipitate probably due to the low surface charge.

### 2.2. Evaluation of FNPs Margination

Once prepared, FNPs were used to study the effects of several parameters on the particle margination, in order to forecast the FNPs transport in microcirculation and to optimize drug delivery strategy for intravascular administration.

In detail, in order to evaluate the effect of RBC volume fraction (i.e., hematocrit (Hct)) on FNP flow and margination, the velocity profile and the radial distribution of FNP/RBC suspensions at Hct 10% and Hct 15% have been estimated by using high-speed video microscopy and image analysis techniques. In particular, the effect of Hct has been investigated by varying the imposed pressure drops (i.e., 20, 40 and 75 mmHg) and by keeping the values of FNP concentration constant at 0.01% by weight (wt).

#### 2.2.1. Velocity Profile

**Figure 2:**
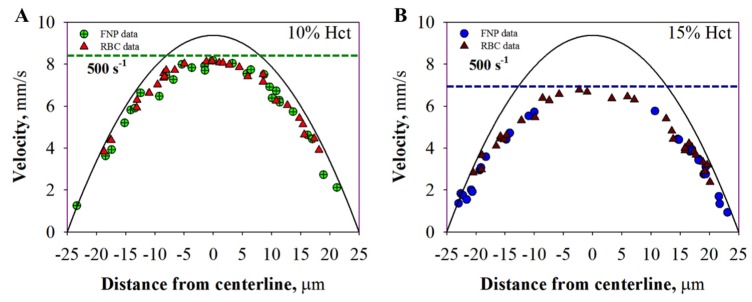
Velocity profile of RBCs (triangles) and FNPs (crossed circles) flowing in a glass microcapillary at 20 mmHg, corresponding to a wall shear rate of 500 s^−1^: (**A**) FNPs (green crossed dots) and RBC (red triangles) experimental velocity profile at Hct 10%; and (**B**) FNPs (blue crossed dots) and RBC (dark red triangles) experimental velocity profile at Hct 15%. (For interpretation of the references to color in this figure legend, the reader is referred to the web version of this article.)

The velocity profiles have been determined as previously reported [3,9,12]. FNP/RBC velocity profiles at 20 mmHg are shown in Figure 2 as a function of the distance from the centerline for the two hematocrit values investigated: Hct 10% (Figure 2A) and Hct 15% (Figure 2B).

In both Figures, each point represents RBC/FNP velocity after curvature correction by using the Snell’s law [3]. The continuous line represents the parabolic profile of the suspending fluid velocity, calculated by applying the Poiseuille’s law at the same pressure drop (i.e., 20 mmHg), with a viscosity of 1 cPoise (i.e., viscosity of the suspending fluid), a tube length of 5 cm and a capillary inner diameter of 50 mm (i.e., with no fitting parameters) [21]. 

As shown in Figure 1A, there is a not negligible difference (green dotted line) between the suspending fluid velocity profile (continuous line) and the experimental data of FNP/RBC (green circles and red triangles) at 10% Hct. This disparity is likely due to the increment of the suspension viscosity brought about by the presence of RBCs. Furthermore, as shown in Figure 2B, a remarkable difference in velocity profiles (blue dotted line) occurred when FNPs were suspended in the fluid at Hct 15%. This reduction is due to the additional increment of the suspension viscosity. In both cases, RBC and FNP velocity profiles superimpose one to each other for a given value of the imposed shear rate: in other words, the velocity distribution of FNPs is governed by the presence of RBCs.

**Figure 3:**
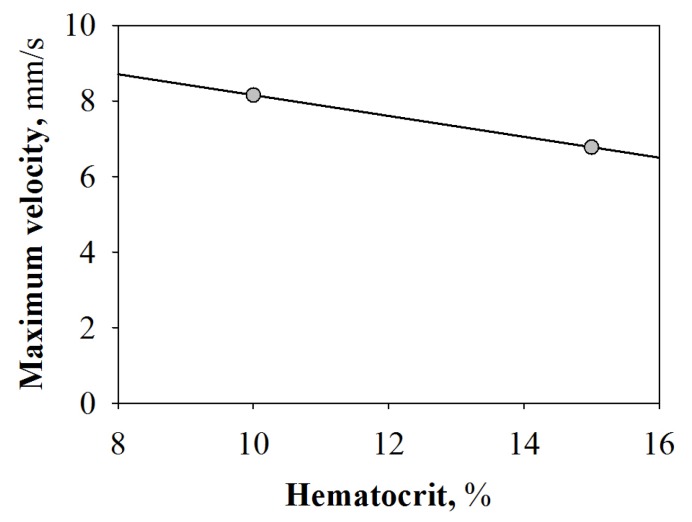
Plot of maximum velocities of FNP/RBC suspensions as a function of Hct at an imposed wall shear.

Thus, as shown in Figure 3, the maximum velocity of FNP/RBC suspensions flowing through a capillary is inversely proportional to the Hct; such fall of the suspension velocity is the consequence of increasing amounts of RBCs and, therefore, of a rise in blood viscosity.

By using the Poiseuille’s law for steady flow condition, an increment of 20% in the suspension viscosity has been estimated. The same trend has been observed for all the pressure drops investigated (data not shown for the sake of brevity and clarity). 

#### 2.2.2. Radial Distribution

**Figure 4:**
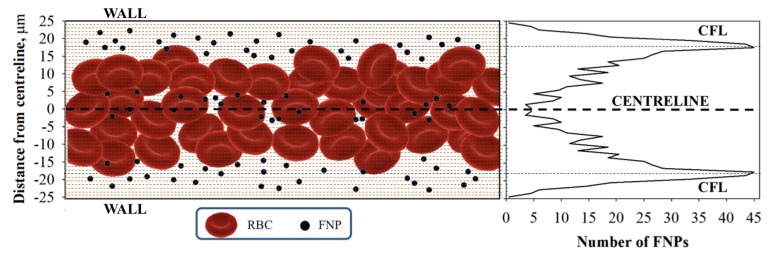
Cartoon of FNP flow and radial distribution in the presence of RBC flow inside the microcapillary.

The radial distributions of FNPs have been determined by using image analysis, according to the methodology described previously [3]. In other words, the number of FNPs present in each layer of the capillary section (i.e., 2% of the center plane) has been averaged between pairs of layers symmetric to the centerline and has been plotted as a function of distance from the centerline (Figure 4).

**Figure 5:**
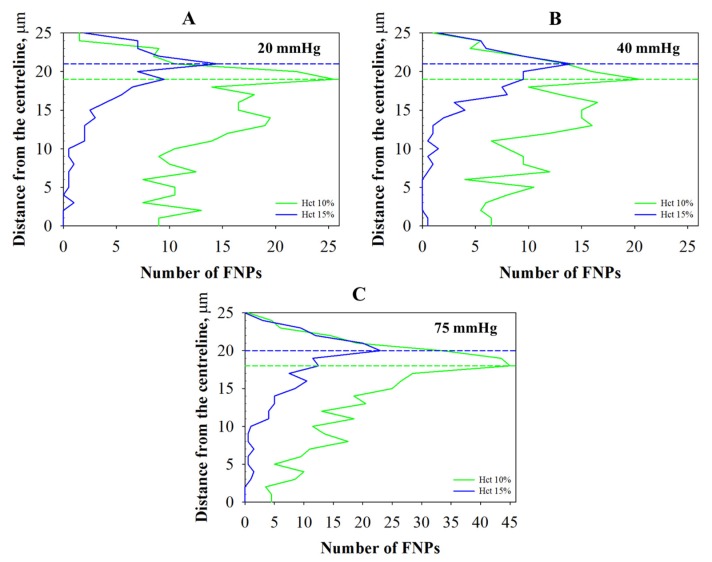
Distribution profiles of FNPs along radius at 10% Hct (green lines) and at 15% Hct (blue lines).FNP radial distribution at: (**A**) 20 mmHg; (**B**) 40 mmHg; and (**C**) 75 mmHg. Dashed lines indicate the cell-free layer (CFL) thickness measured at the corresponding shear rate. (For interpretation of the references to color in this figure legend, the reader is referred to the web version of this article).

FNP radial distribution (half-capillary) is shown in Figure 5 for the pressure drops investigated (i.e., 20, 40 and 75 mmHg) at 10% Hct (green lines) and 15% Hct (blue lines).

As shown in Figure 5, there is a not uniform distribution of FNPs along the radial direction for all the pressure drops investigated, regardless of the Hct. This distribution is due to the collisions between the RBCs and the FNPs travelled across the field of view, that promote a higher number of particles near the wall (i.e., in the CFL) than in the center of the capillary. All the results show a similar trend, but the number of marginated FPNs (i.e., the ones in the CFL) is clearly dependent on Hct and shear rate; the number of FNPs in the outer layers, indeed, increases with the pressure drops and decreased with the RBC concentration. In particular, as shown in previous works, the peaks of distributions in Figure 4 are located at a distance from capillary wall (dotted lines) that corresponds to the edges of the CFL measured at the corresponding shear rate. CFL thickness has been measured by image analysis techniques as the distance between the capillary walls and the outer edge of the RBC core.

**Figure 6:**
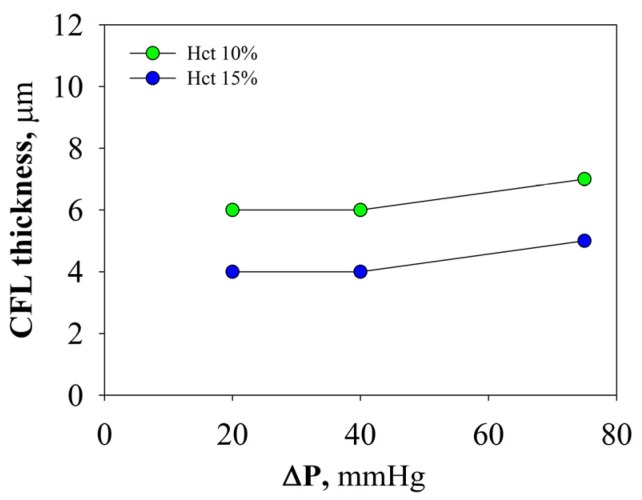
CFL thickness for different shear rates at 10% and 15% Hct. (For interpretation of the references to color in this figure legend, the reader is referred to the web version of this article).

As shown in Figure 6, the average value of the CFL at Hct 10% is about 6 μm at 20/40 mmHg and 7 μm at 75 mmHg, while at Hct 15% it is about 4 μm at 20/40 mmHg and 5 μm at 75 mmHg, finding a very good agreement with the results from numerical simulations and experiments in similar flow conditions [3,9,12,22].

Therefore, the CFL increases when decreasing Hct, because the reduced collisions among RBCs promote their axial migration. The CFL also increases with fast flow that, accelerating the tank-tread motion of cell membrane, facilitates the axial migration of RBCs. 

#### 2.2.3. Margination

**Figure 7:**
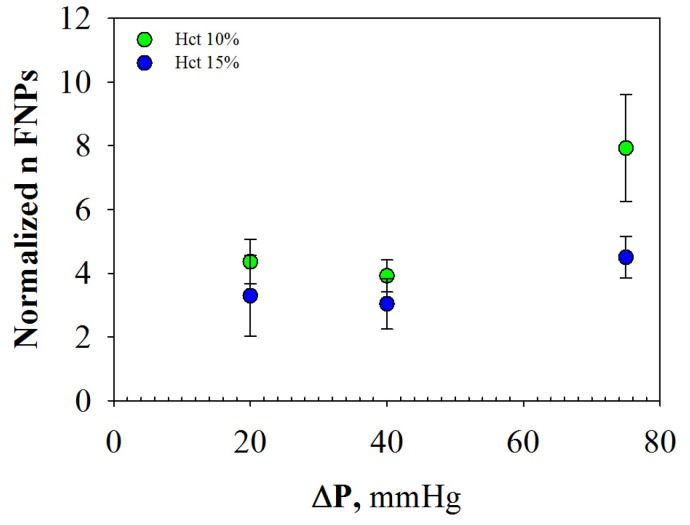
Normalized number of FNPs marginated as a function of shear rate at 10% and 15% Hct. There is a significant difference between the two sets of data at 75 mmHg (*p* = 0.030). (For interpretation of the references to color in this figure legend, the reader is referred to the web version of this article).

To quantify and compare the effect of Hct and shear rate on FNPs margination, the number of marginated particles (i.e., in the CFL) has been examined in dependence on the corresponding CFL thickness. The so-obtained normalized number of FNPs in the CFL has been plotted as a function of pressure drops in Figure 7.

A slightly increasing trend of FNP margination as a function of shear rate is observed for the two Hct values investigated. This result is in line with experimental works on platelet/particle margination as function of shear rate [12,23,24]; at higher shear rate, indeed, the RBC tumbling motion is faster, increasing the probability that FNPs are pushed in the CFL [25,26,27]. Furthermore, margination increased significantly with decreasing Hct; the margination at 75 mmHg, indeed, was significantly lower for the RBC concentration at 15% (statistically significant: *p* = 0.030). This result was in line with numerical and experimental works on WBC/sphere margination, in which it was found that margination propensity increased until a certain Hct value and significantly decreased with a further growth in RBC concentration [28,29,30,31].

### 2.3. Hemocompatibility

**Figure 8:**
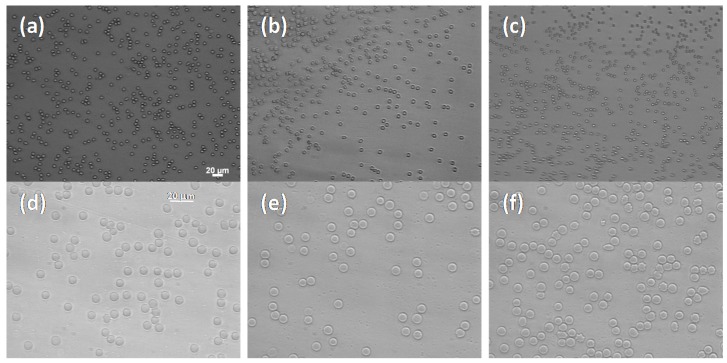
Optical micrographs of: RBC cells alone (**a**,**d**); and in the presence of FNPs (0.01 wt %) at: 10% Hct (**b**,**e**); or 15% Hct (**c**,**f**). Images (**a**–**c**) are at magnification 20×, while (**d**–**f**) are at magnification 40×.

Finally, the in vitro FNPs interaction with erythrocyte membranes and their hemolytic effects on RBC were evaluated at 10% and 15% of Hbt. In fact, hemocompatibility is an essential requirement for successful use of FNPs as in vivo drug carriers, especially after intravenous administration. In particular, hemolysis experiments were carried out by incubating under proper conditions FNPs with erythrocytes (at concentrations used for the margination experiments) for 2 h at 37 °C and quantifying the release of hemoglobin. Erythrocytes were treated with 1 wt % Triton X-100 and PBS at pH 7.4 to obtain the values corresponding to 100% and 0% of lysis, respectively. Under these conditions, FNPs showed no significant hemolytic effects: the percent being of lysis less than 5% (4.8% and 2.7% when incubated at 10% and 15% Hbt, respectively), thus indicating no detectable interaction with RBC membranes. Moreover, no aggregation of erythrocytes was also detected by microscopic observations (Figure 8). 

## 3. Materials and Methods 

### 3.1. Synthesis of PHEA-RhB-PLA-PEG Graft Copolymer and Characterization

α,β-Poly(N-2-hydroxyethyl)-d,l-aspartamide (PHEA) was obtained and characterized as reported in literature [16]. 

The chemical functionalization of PHEA with RhB, PLA and PEG molecules was done by already reported procedures [15,18,19,32], obtaining the PHEA-RhB-PLA-PEG copolymer with a yield of 85 wt % respect to the starting PHEA. Spectroscopic data were in agreement with the reported structure. 

### 3.2. High Pressure Homogenization (HPH) for Nanoparticle Preparation

FNPs were obtained by following an already reported process, the HPH [15,33]. Briefly, a PHEA-RhB-PLA-PEG dispersion in dichloromethane at a concentration of 17 mg/mL (6 mL) was used as organic phase and mixed by stirring at 20,500 rpm with an aqueous phase (50 mL). After dilution by addition of bidistilled water (50 mL), the o/w emulsion was homogenized one time at 7500 psi by using an EmulsiFlex TM-C5 as homogenizer (Avestin Inc., Ottawa, ON, Canada). Evaporation of organic solvent under reduced pressure by using a evaporation system constituted by a water bath B-480, a rotavapor R-114, a Recirculating Chiller F-105 and a Vacuum Controller V-800 (Buchi) allows to obtain FNPs. Finally, each FNP batch was dried by using a Modulyo freeze-dryer (Labconco Corporation, Kansas City, MO 64132, USA) after the addition of PVP as cryoprotectant at a nanoparticle/PVP weight ratio equal to 1:1, and stored as fluffy powder at −20 °C for successive characterization. 

### 3.3. FNPs Characterization: Mean Size and ζ Potential 

The mean number distribution diameter of FNPs were performed by photon correlation spectroscopy (PCS) by using the Zetasizer Nano ZSP (Malvern Instrument). Analyses were done at a fixed angle of 173 °C and at 25 °C by dispersing each sample in Dulbecco’s phosphate buffer saline (DPBS) medium. ζ potential measurement were done by dispersing each sample in DPBS and by analyzing it with the Zetasizer Nano ZSP. 

### 3.4. Blood Samples

Fresh venous blood samples have been drawn from healthy donors and diluted to a concentration of 10% or 15% by volume with ACD (0.6% citric acid, 2.3% sodium citrate, 1.1% anhydrous dextrose and 96% water) as anticoagulant and bovine serum albumin and used within 4 h of collection, following the protocols for blood testing [34]. The concentrations of 10% and 15% have been selected to match the hematocrit value (Hct) in microcirculation, the higher values of Hct (around 45%) found in routine blood tests being associated with large vessels, whereas Hct decreases going from the macro- to the micro-vasculature [35]. This is due to the entrance effects and the inhomogeneous radial distribution of RBCs that tend to concentrate along the vessel centerline (Fahraeus effect) [36].

### 3.5. FNP and RBC/FNP Suspension

Per each experiment, two different suspensions have been used: (i) a FNP/RBC suspension, Hct = 10%, consisting of FNP (0.01 wt %) in a RBC suspension with Hct = 10%; (ii) a FNP/RBC suspension, Hct = 15%, consisting of FNP (0.01 wt %) in a RBC suspension with Hct = 15%. RBC are diluted in ACD and bovine serum albumin (1 g in 5 mL of ACD).

### 3.6. Experimental Set Up

The experiments have been carried out in 50 μm diameter silica microcapillaries (Polymicro Technologies, Phoenix, AZ, USA) placed on a motorized x–y stage (Ludl, Hawthorne, NY, USA) of an inverted microscope (Zeiss Axiovert 100, Milan, Italy), as reported in detail in previous works [3,9]. The FNP/RBC suspensions is fed in the microcapillary tube connected to a μ-pumping system (Fluigent) by which it is possible to impose values of pressure drop in the physiological range ensuring the hydrodynamic conditions found in human microcirculation in vivo, such as laminar flow and physiological pressure (20–60 mmHg) [37]. Images of the flowing suspensions have been acquired with a high-speed video camera (Phantom 4.3, operated up to 1000 frames/s) using a high magnification oil immersion objective (Zeiss 440080 Achroplan 100×/1.25 Oil). A 2D view is ensured by the fact that the images have been acquired focusing on the capillary midplane, around which the velocity profile is rather flat, and that the velocity variations along the vertical direction are quite small in the optical section.

### 3.7. Hemolytic Test and Erythro-Aggregation 

Human RBC were isolated from fresh citrated-treated blood of a healthy voluntary donor, in compliance with the Italian laws and institutional guidelines, and immediately used. In particular, RBC were collected by centrifugation at 1000× *g* for 10 min, washed three times with cold isotonic phosphate saline buffer (PBS) at pH 7.4 and diluted to achieve final concentration (10 or 15%, *v*/*v*) in PBS. This stock dispersion was always freshly prepared and used within 24 h after preparation. A concentrated FNPs dispersion was added to the erythrocyte suspension in order to obtain a final FNPs concentration of 0.04 wt % and incubated for 2 h at 37 °C under constant shaking. After centrifugation, the release of hemoglobin was determined by photometric analysis of the supernatant at 540 nm. Complete hemolysis was achieved by using a 1 wt % aqueous solution of Triton X-100 (100% control value). Each experiment was performed in triplicate and repeated twice. The erythrocyte lysis percentage was calculated according to the following formula:

$$\%\;\mathrm{lysis}=\frac{\left(\begin{array}{ccc} A_{\mathrm{sample}} & - & A_{\mathrm{blank}} \end{array}\right)}{\left(\begin{array}{ccc} A_{100\%\mathrm{lysis}} & - & A_{\mathrm{blank}} \end{array}\right)}\times 100$$

where A_sample_ is the absorbance value of the hemoglobin released from erythrocytes treated with FNPs dispersion; A_blank_ is the absorbance value of the hemoglobin released from erythrocytes treated with PBS buffer; and A_100%lysis_ is the absorbance value of the hemoglobin released from erythrocytes treated with 1% Triton X-100 solution.

Each pellet was diluted with PBS and putted on a microscopic slide. The RBC morphology and aggregation behavior was analyzed using an inverted epifluorescence microscope Zeiss. Five images minimum were taken by Axio Cam MRm (Zeiss) from different parts of the slides and were visually examined.

## 4. Conclusions

In this work, a microfluidic-based experimental set-up coupling microfluidics and microscopy techniques is presented, allowing a quantitative analysis of the influence of RBCs on the transport of micron-sized drug carriers, i.e., fluorescent polymeric nanoparticles (FNP), in vitro. This work mimic the mechanisms that regulate the transport of injectable carriers in the microcirculation, thus elucidating the effect of blood flow parameters and particle characteristics on FNP transport towards the vessel vasculature in micro-capillary flow. In particular, the Fåhræus and Fåhræus–Lindqvist effects promote the RBC confinement in the vessel core during blood flow. FNP, as well as WBCs and platelets, are also distributed non-uniformly in flow but, in opposition to RBCs, they tend to migrate near the blood vessel walls. The results show that margination, which is almost absent when particles are suspended in a cell-free medium, is drastically enhanced by the presence of RBCs. The absence of RBC lysis and/or aggregation in the presence of FNPs at experimental conditions use for the in vitro margination experiment was also demonstrated.

In conclusion, the increase in the margination dynamics is enhanced by the pressure drop: in particular, as the latter increases, the FNP concentration in the RBC-FL increases too, in accordance with previous results on platelet margination [23,24] and independently on RBC volume fraction. Furthermore, the increase of hematocrit leads to a decreasing tendency for FPP margination.

As a concluding remark, this study of margination presents new insights about the dependence of drug carriers margination on blood flow properties (pressure drop) and RBC volume fraction (i.e., hematocrit in the physiological range for microcirculation); moreover, the developed microfluidic approach can be relevant for the design of novel therapeutic system for systemic administration.
